# Supplementary material for: Urine manganese, cadmium, lead, arsenic, and selenium among autism spectrum disorder children in Kuala Lumpur
Source: PeerJ. 2024 Jul 4;12:e17660. doi: 10.7717/peerj.17660 (PMC11227810; doi:10.7717/peerj.17660)
Supplement: Supplemental Information 2 [file peerj-12-17660-s002.docx]

**CODEBOOK FOR SPSS CATEGORICAL DATA**

| **Variable** | **Variables Abbreviation** | **Code** |
| --- | --- | --- |
| Parent Age Classification | parentage30 | 1= Less than 30 years old  2= More than 30 years old |
| Parental Gender | parentSex | 1= Male  2= Female |
| Parental Race | parentrace | 1= Malay  2= Chinese  3= Indian  4= Others |
| Parental Race (Malay vs. non-Malay) | parentrace2 | 0= Nonmalay  1= Malay |
| Parental Education Level | parentedu | 1= Primary Education  2= Secondary Education  3= Tertiary Education |
| Family Income Classification | incomeclass | 0= M40T20  1= B40 |
| Stay Outside KL | residentarea | 1= Inside KL  2= Outside KL |
| Children Age Group | kidage4 | 1= 4 years old or less  2= More than 4 years old |
| Children Gender | kidgender | 1= Male  2= Female |
| Children Gender | kidgender2 | 0= Female  1= Male |
| History of ASD among Siblings | familyasd | 0= No  1= Yes |
| Hospital of Birth | Kidbirthhosp | 1= Government  2= Private |
| State of Birth | Kidbirthstate | 1= Kuala Lumpur  2= Selangor  3= Others |
| Prematurity | Kidprem | 0= No prematurity  1= Prematurity |
| Baby's Complication | Kidcompli | 0= No  1= Yes |
| Breastfeeding | bfeed | 0= No  1= Yes |
| Status of Immunisation | Immunstatus | 1= Up to date  2= Missing |
| Stage of ASD | autismstage | 1= Mild autism  2= Moderate autism  3= No autism |
| Speak at Three Years Old | kidspeak | 1= Yes  2= No |
| Advanced Maternal Age | advmatage | 1= Less than 35 years old  2= More than 35 years old |
| Mode of Delivery | modedeliv | 1= Spontaneous Vertex  2= Assisted Delivery  3= Caesarean Section |
| Hemoglobin Level | Anemiapreg11 | 1= 11.0g/dL or less  2= More than 11.0g/dL |
| Gestational Diabetes Melitus | Gdm | 1= Yes  2= No |
| Pregnancy Induced Hypertension | Pih | 1= Yes  2= No |
| Other Comorbidity During Pregnancy | comorbidpreg | 1= Yes  2= No |
| Other Kids Having Autism Spectrum Disorder | othkidautism | 1= Yes  2= No |
| Type of House | housetype | 1= Bungalow  2= Semi detached  3= Terrace  4= Town House  5= Condominium  6= Apartment  7= Flat |
| Classification of House's Age | Agehousclas25 | 1= Yes  2= No |
| House Nearby the Main Road | houseroad | 1= Yes  2= No |
| House Nearby the Factory | housefactory | 1= Yes  2= No |
| Source of Drinking Water from Tap Water | drinkwater | 1= Yes  2= No |
| Parental Smoking Status | parentsmokestat | 0= Nonsmoker  1= Active Smoker  2= Ex smoker |
| Risk at workplace | riskworkplace | 0= norisk  1= yesrisk |
| Exposure to the Soil | soilexpos | 1= Everyday  2= Once a week  3= Once a month  4= Never |
| Sucking Own Hand | suckhand | 1= All the time  2= Just before sleep  3= Never |
| PICA (Eat Things Other than Food) | pica | 1= Yes  2= No  3= Not sure |
| Washing Children Hand | washhand | 1= Every time after hand contact  1= Before eating and while taking bath  2= While taking bath |
| Supplement with Vitamin | supplemenvit | 1= Yes  2= No |
| Drinking the Milk | drinkmilk | 1= More than 2 times per day  2= 1 to 2 times per day  3= Never |
| Eating Fruits and Vegetables | eatfruitvege | 1= Every Meal  2= Once a Day  3= Once a Week  4= Never |
| Eating Meat | eatmeat | 1= Every Meal  2= Once a Day  3= Once a Week  4= Never |
| Risk of Exposure at Work Place | exposework | 1= Yes  2= No  3= Not sure |
| birth order category | paritycat | 0= Subsequent child  1= First Child |
| birth weight category | birthweightcat | 0= More than 4.0kg  1= 2.5kg to 4.0kg  2= More than 4.0kg |
